# Supplementary material for: Cells Responding to Closely Related Cholesterol-Dependent Cytolysins Release Extracellular Vesicles with a Common Proteomic Content Including Membrane Repair Proteins
Source: Toxins (Basel). 2022 Dec 20;15(1):4. doi: 10.3390/toxins15010004 (PMC9865450; doi:10.3390/toxins15010004)
Supplement: Supplementary file 1 [file toxins-15-00004-s001.zip › Supplementary figures and legends 1011.pdf]

Supplementary Materials: “Cells Responding to Closely Related Cholesterol-Dependent Cytolysins Release Extracellular Vesicles with a Common Proteomic Content including Membrane Repair Proteins”

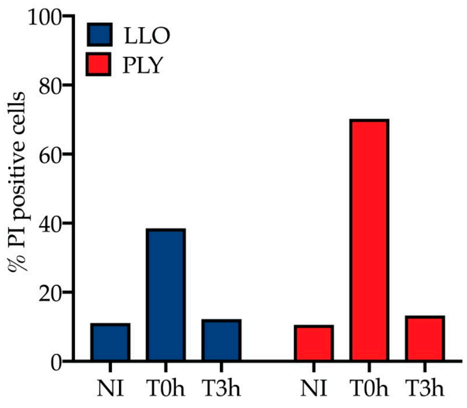

Figure S1. Cells recover full PM integrity 3 h after PFTs washout. Cells were left non-intoxicated (NI) or were intoxicated with LLO (1 nM, blue bars) or PLY (0.2 nM, red bars) for 15 min. PM permeability was measured immediately after 15 min of intoxication (T 0 h) and after 3 h of PFTs washout (T 3 h), by flow cytometry following incorporation of the membrane impermeable dye propidium iodide (PI). Graph shows representative data from a single experiment.

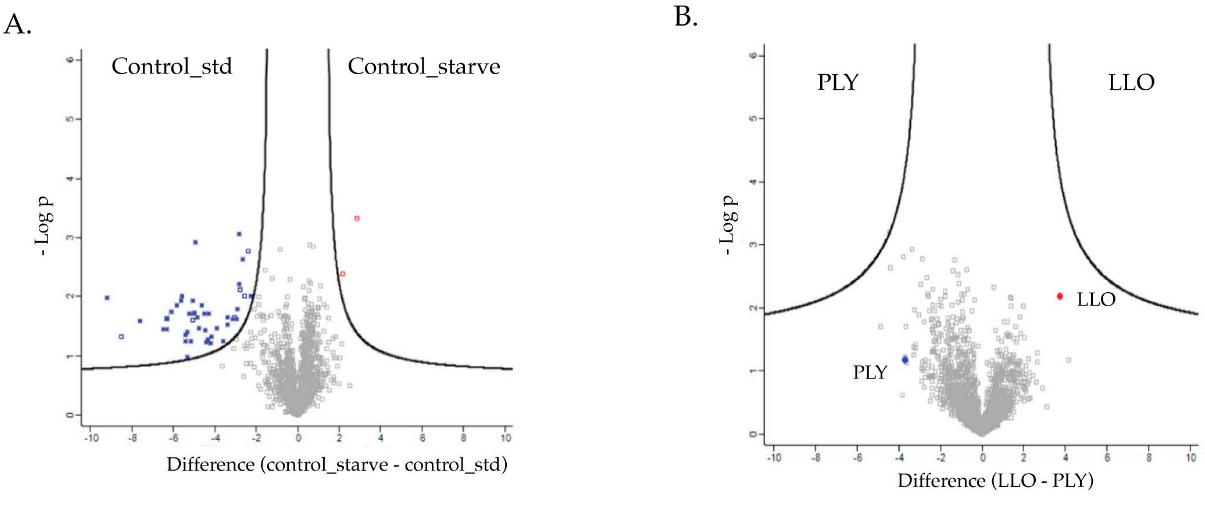

Figure S2. Volcano plots showing the protein levels (x axis), represented in fold change (in log2), detected on A) EVs released from control HeLa cells compared to EVs collected from control starved HeLa cells and B) EVs released from PLY-intoxicated HeLa cells compared to EVs collected from LLO-intoxicated HeLa cells. Three independent experiments were analysed and Student t-tests were applied to calculate -log p values for each protein (y axis). The curved black lines represent the threshold for statistical significance determined by t-test (FDR=0.05 and S0=1). Blue and red squares correspond to overrepresented proteins in the specific condition.

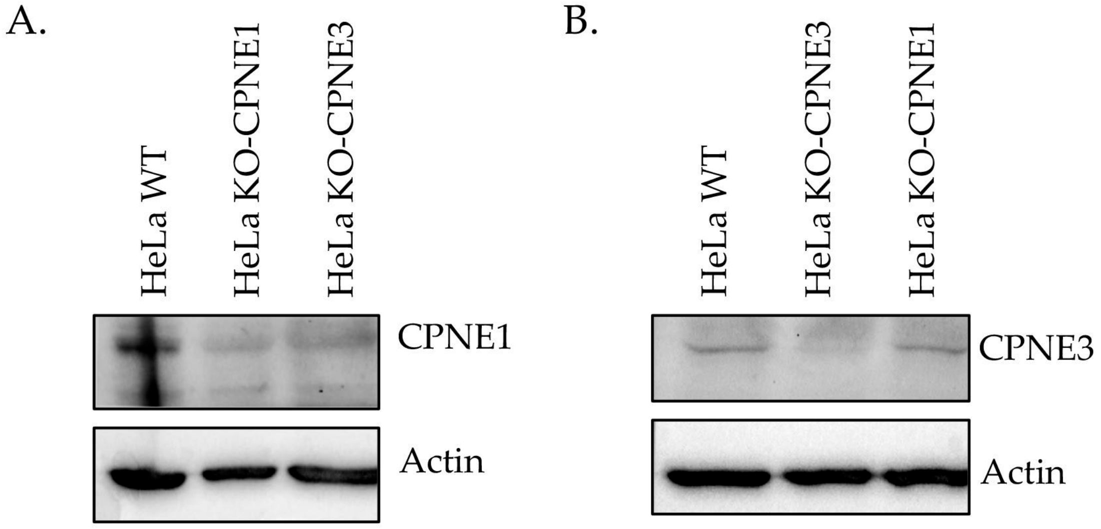

Figure S3. Immunoblot showing the levels of (A) CPNE1 and (B) CPNE3 in WT, KO-CPNE1 and KO-CPNE3 HeLa cells. Total protein extracts were collected from WT, KO-CPNE1 and KO-CPNE3 HeLa cells, western blot analysis was performed, and membranes were stained using (A) CPNE1 or (B) CPNE3 antibody. Actin was used as loading control.

Supplementary Tables. Tables provide proteomics data. **Table S1.** List of proteins detected and reliably quantified in EVs released from control HeLa cells, LLO and PLY-intoxicated cells. **Table S2.** List of proteins showing significantly different abundances in EVs released by control and control starved HeLa cells. **Table S3.** List of proteins showing significantly different abundances in EVs released from PLY-intoxicated and LLO-intoxicated HeLa cells. **Table S4.** List of proteins overrepresented in EVs released by LLO-intoxicated HeLa as compared to control EVs. **Table S5.** List of proteins overrepresented in EVs released by PLY-intoxicated HeLa cells as compared to control EVs. **Table S6.** List of proteins showing significantly different abundances in EVs released from both LLO and PLY-intoxicated HeLa cells as compared to EVs from control cells.

Table S7. Sequence of the oligonucleotides designed as gRNA for CRISPR/Cas-9 gene editing and used for the cloning of CPNE1 and CPNE3 into pEGFP-C1.

**CPNE1 exon 4 (gRNA)**	Forward	5′ CAC CGT TGG CCG GAC TGA ACG GGT G 3′
	Reverse	5′ AAA CCA CCC GTT CAG TCC GGC CAA 3′
**CPNE1 exon 10 (gRNA)**	Forward	5′ CAC CGT TTT CTG CTG CTT CTC AGG G 3′
	Reverse	5′ AAA CCC CTG AGA AGC AGC AGA AAA C 3′
**CPNE3 exon 4 (gRNA)**	Forward	5′ CAC CGA GTG ATG ATG ACT TCT TAG 3′
	Reverse	5′ AAA CCT AAG AAG TCA TCA TCA CTC 3′
**CPNE3 exon 13 (gRNA)**	Forward	5′ CAC CGC GCT CAG ATA CCT CCT CAG 3′
	Reverse	5′ AAA CCT GAG GAG GTA TCT GAG CGC 3′
**GFP-CPNE1 (cloning in pEGFP-C1)**	Forward	5′ATT CTC GAG ACG CCC ACT GCG TGA CCT TGG 3′
	Reverse	5′ATT GTC GAC CTA GGC CTG GGG GGC CTG TG 3′
**GFP-CPNE3 (cloning in pEGFP-C1)**	Forward	5′ATA CTC GAG CAG CTG CCC AGT GTG TCA C 3′
	Reverse	5′TCC GGA TCC TCA CTG CTT CTG TTG TTT CGT GGC 3′

Video S1. Representative video recorded in a NanoSight NS300 system allowing the analysis of the EVs purified samples. EVs appear as bright dots moving following a Brownian movement. The size distribution profile and the concentration of EVs was obtained from this analysis. Here are shown EVs collected from supernatants of non-intoxicated control cells.

Video S2. Representative video recorded in a NanoSight NS300 system allowing the analysis of the EVs purified samples. EVs appear as bright dots moving following a Brownian movement. The size distribution profile and the concentration of EVs was obtained from this analysis. Here are shown EVs collected from supernatants of LLO-intoxicated cells.

Video S3. Representative video recorded in a NanoSight NS300 system allowing the analysis of the EVs purified samples. EVs appear as bright dots moving following the Brownian movement. The size distribution profile and the concentration of EVs was obtained from this analysis. Here are shown EVs collected from supernatants of PLY-intoxicated cells.
