# Supplementary material for: Cells Responding to Closely Related Cholesterol-Dependent Cytolysins Release Extracellular Vesicles with a Common Proteomic Content Including Membrane Repair Proteins
Source: Toxins (Basel). 2022 Dec 20;15(1):4. doi: 10.3390/toxins15010004 (PMC9865450; doi:10.3390/toxins15010004)
Supplement: Supplementary file 1 [file toxins-15-00004-s001.zip › Table S6.pdf]

Table S6. Sequence of the oligonucleotides designed as gRNA for CRISPR/Cas-9 gene editing and used for the cloning of CPNE1 and CPNE3 into pEGFP-C1.

**CPNE1 exon 4 (gRNA)**	Forward	5´CAC CGT TGG CCG GAC TGA ACG GGT G 3´
	Reverse	5´AAA CCA CCC GTT CAG TCC GGC CAA 3´
**CPNE1 exon 10 (gRNA)**	Forward	5´CAC CGT TTT CTG CTG CTT CTC AGG G 3´
	Reverse	5´AAA CCC CTG AGA AGC AGC AGA AAA C 3´
**CPNE3 exon 4 (gRNA)**	Forward	5´CAC CGA GTG ATG ATG ACT TCT TAG 3´
	Reverse	5´AAA CCT AAG AAG TCA TCA TCA CTC 3´
**CPNE3 exon 13 (gRNA)**	Forward	5´CAC CGC GCT CAG ATA CCT CCT CAG 3´
	Reverse	5´AAA CCT GAG GAG GTA TCT GAG CGC 3´
**GFP-CPNE1 (cloning in pEGFP-C1)**	Forward	5´ATT CTC GAG ACG CCC ACT GCG TGA CCT TGG 3´
	Reverse	5´ATT GTC GAC CTA GGC CTG GGG GGC CTG TG 3´
**GFP-CPNE3 (cloning in pEGFP-C1)**	Forward	5´ATA CTC GAG CAG CTG CCC AGT GTG TCA C 3´
	Reverse	5´TCC GGA TCC TCA CTG CTT CTG TTG TTT CGT GGC 3´
