# Supplementary figures and images for: Cells Responding to Closely Related Cholesterol-Dependent Cytolysins Release Extracellular Vesicles with a Common Proteomic Content Including Membrane Repair Proteins
Source: Toxins (Basel). 2022 Dec 20;15(1):4. doi: 10.3390/toxins15010004 (PMC9865450; doi:10.3390/toxins15010004)

Related to figure 4D

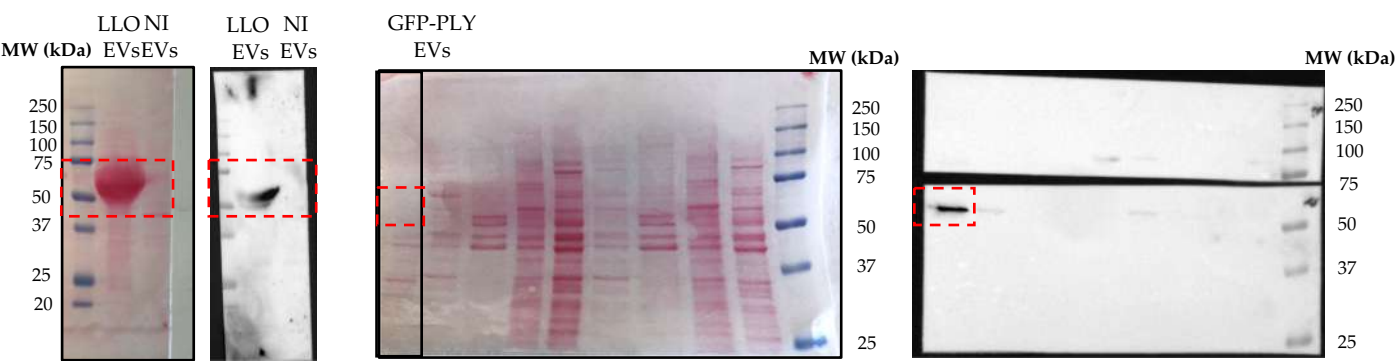

Related to supplementary figure S3

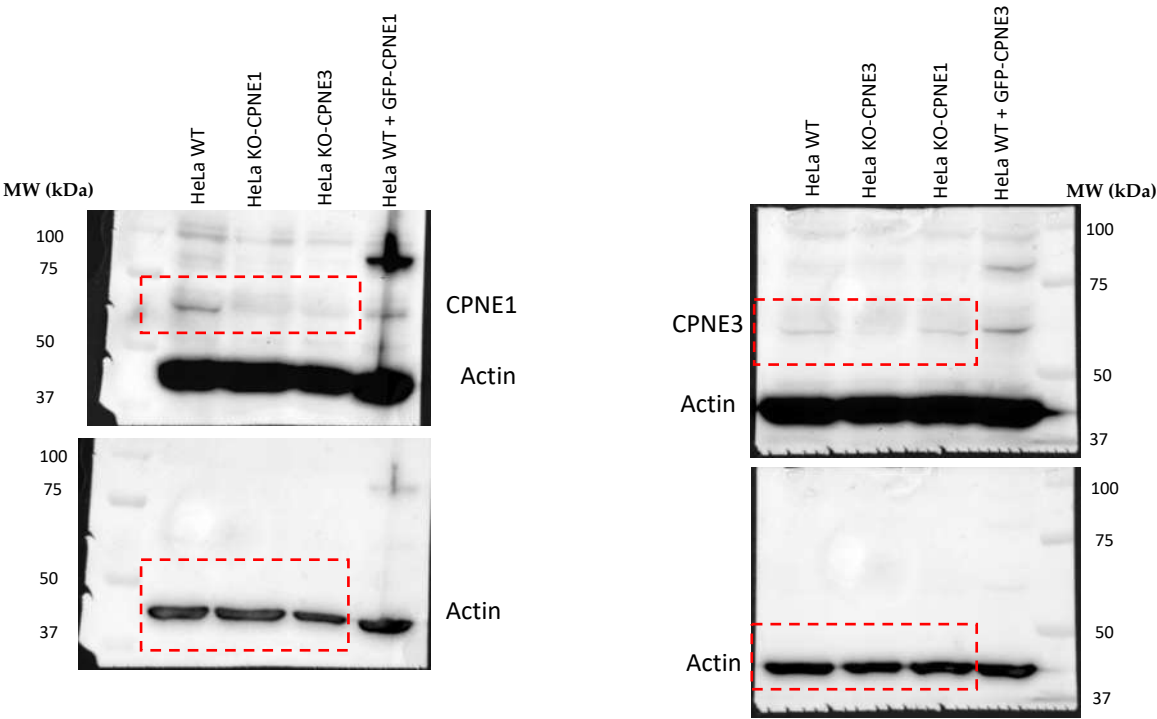

Supplement: Supplementary file 1 [file toxins-15-00004-s001.zip › Blots_SupFiles_compressed.pdf]
